# Benefits of a Supervised Ambulatory Outpatient Program in a Cardiovascular Rehabilitation Unit Prior to a Heart Transplant: A Case Study

**DOI:** 10.3389/fcvm.2022.811458

**Published:** 2022-05-19

**Authors:** Antoine Poty, Florent Krim, Philippe Lopes, Yves Garaud, Pierre-Marie Leprêtre

**Affiliations:** ^1^Exercise Physiology and Rehabilitation Laboratory, Picardie Jules Verne University, Amiens, France; ^2^Fundation Léopold Bellan, Chateau d’Ollencourt, Unit of Cardiac Rehabilitation, Tracy-le-Mont, France; ^3^Service de Réadaptation Cardiovasculaire, Centre Hospitalier de Corbie, Corbie, France; ^4^Association Picardie de Recherche en Réadaptation Cardiaque, Association Picardie de Recherche en Réadaptation Cardiaque, Corbie, France; ^5^Laboratoire de Biologie de l’Exercice Pour la Performance et la Santé, Université d’Évry Val d’Essonne, Évry, France

**Keywords:** transplant, preoperative aerobic capacity, rehabilitation, vital prognostic, cardiac patient

## Abstract

Preoperative peak oxygen uptake ($\dot{\text{V}}$O_2p*eak*_) and ventilatory efficiency ($\dot{\text{V}}$_*E*_/$\dot{\text{V}}$CO_2_slope) are related to the vital prognosis after cardiac transplantation (HTx). The objective of our study was to evaluate the effects of exercise-based cardiac rehabilitation (ECR) program on the preoperative exercise capacity of a HTx candidate. A male patient, aged 50–55 years, with chronic heart failure was placed on the HTx list and performed 12 weeks of intensive ECR (5 sessions-a-week). Our results showed that the cardiac index continuously increased between the onset and the end of ECR (1.40 vs. 2.53 L.min^–1^.m^2^). The first 20 sessions of ECR induced a $\dot{\text{V}}$O_2p*eak*_ increase (15.0 vs. 19.3 ml.min^–1^.kg^–1^, corresponding to 42.0 and 53.0% of its maximal predicted values, respectively). The peak $\dot{\text{V}}$O_2_ plateaued between the 20th and the 40th ECR session (19.3 vs. 19.4 ml.min^–1^.kg^–1^) then progressively increased until the 60th ECR session to reach 25.7 ml.min^–1^.kg^–1^, i.e., 71.0% of the maximal predicted values. The slope of $\dot{\text{V}}$_E_/$\dot{\text{V}}$CO_2_ showed a biphasic response during the ECR program, with an increase between the onset and the 20th ECR session (58.02 vs. 70.48) and a decrease between the 20th and the 40th ECR session (70.48 vs. 40.94) to reach its minimal value at the 60th ECR session (31.97). After the first 40 sessions of the ECR program, the Seattle Heart Failure Model score predicted median survival time was estimated at 7.2 years. In conclusion, the improvement in exercise capacity and cardiorespiratory function following the ECR helped delay the heart transplant surgery in our patient awaiting heart transplantation.

## Introduction

Cardiac transplantation is the treatment of choice for many patients with end-stage heart failure who remain symptomatic despite optimal medical therapy. For carefully selected patients, heart transplantation offers markedly improved survival and quality of life (1). Khush et al. reported that the functional status after heart transplantation remained very good compared to living with advanced heart failure. Indeed, over 70% of recipients could perform normal daily life activities with no or minimal symptoms (2). Post-transplant survival also improved over time. The median survival time after adult heart transplants increased from 12.5 years in 2002 to 14.8 years in 2009 among 1-year survivors (2). However, comorbidities in patients with heart failure, like arterial high blood pressure, lung and kidney damage, or chronic obstructive pulmonary disease (COPD) raised the question of the transplantation procedure and the long-term patient’s outcome. Additionally, heart transplantation requires a sufficient number of donors, the induction and the maintenance of immunosuppressants, lipid-lowering medication, and vasodilators used in cardiac disease, which diminish peak oxygen uptake ($\dot{\text{V}}$O_2p*eak*_) (3–5). The $\dot{\text{V}}$O_2p*eak*_, expressed in absolute or in% of the theoretical maximal value of the patient, is also used for heart transplantation referral (6, 7). Patients showing a $\dot{\text{V}}$O_2p*eak*_ lower than 50% of predicted should be seriously considered for transplantation (8). Similarly, patients showing a $\dot{\text{V}}$O_2p*eak*_ greater than 60% of predicted should not warrant listing as transplant candidates in the absence of other significant risk factors, including unresponsiveness to drug therapy (8, 9).

Non-pharmacological strategies have been recommended to improve exercise capacity at the preoperative stage (10). Prior cardiac surgery and the use of implanted devices were associated with increased 1- and 5-year mortality (2). Fonarow et al. also found that the combination of optimized medical therapy and regular physical activity performed below the anaerobic threshold (AnTh) induced an increase in $\dot{\text{V}}$O_2p*eak*_ in heart transplant candidates (11). Namely, optimized medical therapy and regular physical activity reduced hospital admission rate in patients who led to complete 6 months of follow-up and a significant increase in functional capacity responses: 49% of 179 patients have been downgraded from New York Heart Association (NYHA) class III or IV before referral to I or II after 6 months of follow-up (11). This conclusion should be taken with caution because the results depended on the patient care model. Cardiac rehabilitation programs are aimed at limiting the psychological and physiological stresses of cardiovascular disease, optimizing cardiovascular risk reduction, reducing disability, and improving cardiovascular function to achieve the highest quality of life possible in patients (12). However, Leprêtre et al. (13) emphasized that the numerous models of care for heart disease could explain the low-level evidence for the beneficial effects of ECR (14). Moreover, ECR is, to our knowledge, mostly proposed in secondary prevention for heart transplant candidates (15). To our knowledge, only Gimeno-Santos et al. showed beneficial effects on functional and exercise capacity in patients waiting for heart transplantation (16).

It has been established that preoperative exercise capacity is a predictor of vital prognosis after transplantation in patients with cardiac insufficiency (17). Reduced exercise tolerance observed in chronic heart failure was mainly attributed to impaired skeletal muscle function (18, 19), which was indirectly evaluated by ventilatory efficiency, i.e., the slope of $\dot{\text{V}}$_E_/$\dot{\text{V}}$CO_2_ slope, during a cardiopulmonary exercise test (CPET) (20). Hence, the $\dot{\text{V}}$_E_/$\dot{\text{V}}$CO_2_ slope is an additional parameter that has to be considered together with $\dot{\text{V}}$O_2p*eak*_, for estimating the exercise capacity and patient outcome (21, 22). Thus, the objective of this study was to evaluate the effects of an exercise-based cardiac rehabilitation program on the preoperative exercise capacity of a heart transplant candidate, evaluated from $\dot{\text{V}}$O_2p*eak*_ and the ventilatory efficiency. We hypothesized that a short exercise program based on cardiac rehabilitation could delay heart transplantation by improving prognosis with an increased functional capacity in a patient with end-stage heart failure.

## Methods

### Subject

A male patient, aged 50–55 years and placed on a heart transplantation list, was admitted for full hospitalization to perform a cardiac rehabilitation exercise program following seven heart failure relapses during the first half of 2018. This dyslipidemic patient with chronic obstructive pulmonary disease (NYHA class II dyspnea), had, at the beginning of the exercise-based cardiac rehabilitation program, severe ischemic heart disease with a dilated and hypertrophic left ventricle (LV), a bi-ventricular dysfunction (Tricuspid annular plane systolic excursion, i.e., TAPSE: 6.0 mm) with impaired right ventricular systolic function, moderate grade 2 mitral insufficiency, a significant grade 3 diastolic dysfunction, and a chronic tendency to hypotension (systolic blood pressure ≃ 80–90 mmHg). These complications followed a myocardial infarction that occurred 5 years previously resulting in the insertion of a triple-chamber defibrillator, three stents, and a resynchronization. At presentation, he was a sedentary former smoker and former drinker. The Seattle Heart Failure Model (SHFM) score predicted a median survival time of 1.5 years. His height, weight, and body mass index (BMI) were 180 cm, 61.0 kg, and 18.8, respectively. His medication consisted of a diuretic (125 mg.day^–1^), an aldosterone inhibitor (50 mg.day^–1^), a beta-blocker (3.75 mg.day^–1^), a hyper polarization-activated cyclic nucleotide-gated (HCN) inhibitor (5 mg, twice a day), and Entresto^®^ (24/26 mg, two times daily). Before participation, the subject was informed of the risks and discomforts associated with the protocol and gave his written informed consent in accordance with the local ethics committee approval and the Ethical Standards in Sport and Exercise Science Research published in the International Journal of Sports Medicine (23). Written informed consent was obtained from the patient for the publication of any potentially identifiable data included in this article according to the CARE guidelines (24).

### Protocol

A resting cardiac ultrasound (Vivid 9 Digital Ultrasound System Echocardiograph, General Electric Health Company, Boston, United States) and an incremental exercise test with gas exchange measurements were performed before (T_0_), as well as after 20 (T_1_), 40 (T_2_), and 60 (T_3_) sessions of exercise-based cardiac rehabilitation. LV volume and left ventricular ejection fraction (LVEF) were calculated using classical methods previously described by Shah et al. (25). The right ventricle contractile reserve was measured by changes in the tricuspid annular plane systolic excursion (TAPSE) (26). The cardiopulmonary exercise test (CPET) was performed on a bicycle ergometer (Ergoselect 200, Ergoline GmbH, Bitz, Germany) using a ramp protocol with increments of 10 w.min^–1^ which was followed by a 3-min loaded pedaling cool down and a 2-min passive recovery in sitting position. Oxygen uptake ($\dot{\text{V}}$O_2_), carbon dioxide production ($\dot{\text{V}}$CO_2_), and minute ventilation ($\dot{\text{V}}$_E_) were measured using a breath-to-breath gas analyzer (Jaeger Vyntus^®^ CPX, Carefusion, Hoechberg, Germany). The $\dot{\text{V}}$O_2_peak was defined as the highest oxygen up takeover in any 15-s period, and the ventilatory efficiency was obtained from the $\dot{\text{V}}$_E_/$\dot{\text{V}}$CO_2_ slope (27). These values were expressed using the international unit system and as a percentage of maximal predictive values (28). Finally, the anaerobic threshold (AnTh) was determined as the breakpoint of the $\dot{\text{V}}$CO_2_curve against $\dot{\text{V}}$O_2_ plot (V-slope method) and expressed in absolute and in percentage of measured peak values (29). A 10-ml venous blood sample was drawn into an EDTA tube to assay plasma N-terminal fragment of the pro-brain natriuretic peptide (NT-proBNP) and to estimate natremia and glomerular filtration rate based on creatinine and patient characteristics (GFR by MDRD) (30, 31). The patient prognosis was assessed by the SHFM score (32).

### Exercise-Based Cardiac Rehabilitation

The cardiac rehabilitation program followed the usual care recommendations (33, 34). The training sessions consisted of a 30-min continuous cycling exercise (Ergoselect 200, Ergoline GmbH, Bitz, Germany) at a steady power output equivalent to 100% of AnTh on 5 days per week. The patient was instructed to maintain a 60-rpm pedaling rate. Each cardiac rehabilitation session was complemented by a 30-min health therapy education, 30-min stretching exercises on the floor, and 60-min walking, 5 days a week, in accordance with current French society of cardiology standards (33). Exercise intensity was adjusted according to the CPET results conducted every 4 weeks.

## Results

**FIGURE 1:**
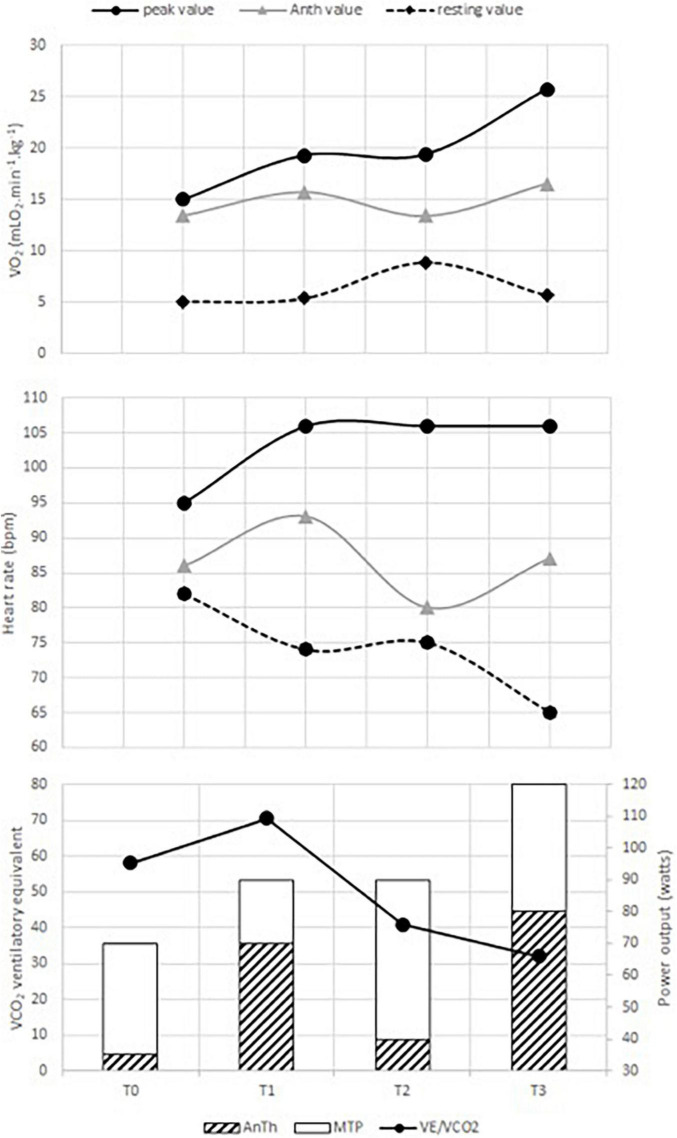
Changes in cardiorespiratory responses induced by the exercise-based cardiac rehabilitation (ECR) program. AnTh is the acronym for anaerobic threshold. $\dot{\text{V}}$O_2_, V_E_/VCO_2_, HR, and MTP represent oxygen uptake, ventilatory equivalent to CO_2_, heart rate, and maximal tolerated power, respectively. T_0_ is the time of the initial evaluation, T_1_ and T_2_, the times of the intermediate evaluation (after the first 20 and 40 sessions), and T_3_, the time of the final evaluation.

Medical treatment did not change during the intervention period. Table 1 presents the data on resting cardiac parameters and renal function. The baseline cardiac index was almost two times lower than the normal value. During the ECR program, the cardiac index increased from 1.40 to 2.53 L.min^–1^.m^–2^after 60 ECR sessions. However, LV remained dilated (92 vs. 88 mm) with an altered ejection fraction—visually assessed at 15 and 20%, at the onset and at the end of ECR program, respectively. TAPSE also increased from 6.0 to 17.8 mm, which approached the normal values in adults (i.e., 18.0 mm) at the end of the ECR program. NT-proBNP (T_0_: 1,267 vs. T_3_: 877 pg.ml^–1^), GFR by MDRD (T_0_: 100.59 vs. T_3_: 92.04 ml.min^–1^), and natremia (T_0_: 136 vs. T_3_: 140 mmol.L^–1^) changes induced by the ECR program showed that the renal function was preserved throughout the whole ECR program. Figure 1 illustrates the cardiorespiratory responses to the ECR. At baseline, $\dot{\text{V}}$O_2_peak and maximal heart rate (HR) were 15.0 ml.min^–1^.kg^–1^ and 95 beats.min^–1^, which corresponded to 42.0 and 57.0% of the maximum predicted values, respectively. These peak values were reached at a maximal tolerated power (MTP) equal to 70 w (i.e., 42.0% of the predicted value). The first 20 sessions of ECR (between T_0_ and T_1_) induced an increase in MTP (70 vs. 90w), $\dot{\text{V}}$O_2p*eak*_ (15.0 vs. 19.3 ml.min^–1^.kg^–1^), and peak HR (95 vs. 106 beats.min^–1^) reaching 54.0% of MTP and $\dot{\text{V}}$O_2p*eak*_ predicted values and 63.0% of maximal theoretical HR at T_1_. Then, the cardiorespiratory measurements remained stable between the 20th and the 40th ECR session ($\dot{\text{V}}$O_2p*eak*_: 19.3 vs. 19.4 ml.min^–1^.kg^–1^, maximal HR: 106 vs. 106 beats.min^–1^ and MTP: 90 vs. 90 W, for T_1_ and T_2_, respectively). Thereafter, the $\dot{\text{V}}$O_2p*eak*_ progressively increased until the end of ECR to reach 25.7 ml.min^–1^.kg^–1^ at T_3_, corresponding to 71.0% of the predicted value. The MTP was 120 W, which was also consistent, reaching 71.0% of the maximal predicted power value. Additionally, the ECR program induced a decrease in resting HR and an improvement in cardiorespiratory parameters with a decrease in AnTh. Consequently, baseline $\dot{\text{V}}$O_2p*eak*_ and MTP were slightly lower than the $\dot{\text{V}}$O_2_, and the mechanical power associated with AnTh was determined at the end of the ECR program. The slope of $\dot{\text{V}}$_E_/$\dot{\text{V}}$CO_2_ showed a biphasic response following the ECR program, with an increase between the baseline and the 20th ECR session (58.02 vs. 70.48 for T_0_ and T_1_, respectively) then the slope of $\dot{\text{V}}$_E_/$\dot{\text{V}}$CO_2_ decreased between T_1_ and the 40th ECR session (70.48 vs. 40.94, T_1_ and T_2_, respectively) to show its lowest value after 60 ECR sessions (T_3_: 31.97). Finally, the SHFM predicted median survival time increased from 1.5 to 7.2 years at the end of the 40 sessions of ECR (i.e., T_2_).

**TABLE 1:** Changes in renal function and resting cardiac parameters induced by the ECR program.

	Normal values	T_0_	T_1_	T_2_	T_3_
Predicted median survival time (SHFM score)	15.2	1.5			7.2
**Echocardiographic parameters**					
Cardiac index, L.min^–1^.m^–2^	3.2–3.8	1.40	1.69	2.44	2.53
LVEF,%	62.8 ± 4.8	15	15	20	20
LV end-diastolic volume, mL	104.2 ± 25.1	240.0	309	366.7	222.7
LV end-systolic volume, mL	38.8 ± 11.2	204.0	262.7	290.4	179.4
LV dilatation, mm	42–58	92	120	87	88
LA area, cm^2^	16.5 ± 3.2	51.2	36.3	29.5	36.0
LA volume, mL	52.5 ± 14.4	116	74.7	66.8	74.1
RA area, cm^2^	14.5 ± 3.2	27.0	12.4	17.0	12.3
RA volume, mL	44.5 ± 15.6	61.2	28.2	38.6	38.0
RV end-diastolic area, cm^2^	18.1 ± 3.9	–	–	–	–
RV end-systolic area, cm^2^	10.1 ± 3.0	–	–	–	–
TAPSE, mm	>16	6.0	17.8	11.4	17.8
**Blood data**					
Hemoglobin, g.dL^–1^	13–18	11.8	11.8	13.5	13.9
NT-proBNP, pg.mL^–1^	<125	1,267	861	689	877
GFR by MDRD, mL.min^–1^.1.73 m^2^	≥90	100.59	74.76	84.76	92.04
Natremia, mmol.L^–1^	136–145	136	134	140	140

*LV and RV are left and right ventricles, respectively. LA and RA are left and right atriums, respectively. LVEF is the acronym for the left ventricular ejection fraction, and TAPSE is the acronym for the tricuspid annular plane systolic excursion. NT-proBNP represents the N-terminal fragment of the pro-brain natriuretic peptide, and GFR by MDRD represents the estimated glomerular filtration rate based on creatinine and patient characteristics (age, race, gender, and plasma creatinine). SHFM is the acronym for the Seattle Heart Failure Model. Normal values corresponded to echocardiographic reference ranges published by Cohen and Soulat-Dufour (54) and in the NORRE study (55).*

## Discussion

Heart transplantation is an established therapy for end-stage heart failure that aims to improve the survival and quality of life of transplant recipients (1, 35). However, challenges still exist. First, the number of patients referred for heart transplantation continues to increase at a rate well beyond the number of available donors (9). Additionally, some complications such as impaired skeletal muscle function or organ rejection, occur (5, 35, 36). Some patients with heart transplant also present physical exercise intolerance. In their review, McMahon et al. (37) reported that $\dot{\text{V}}$O_2_peak remained 70% lower in transplanted patients compared to age-matched controls. The reduced $\dot{\text{V}}$O_2_peak may be related to heart and locomotion skeletal muscle dysfunction (metabolic abnormalities and atrophy) as a result of heart failure (36). Meta-analysis studies provided evidence that ECR benefits patients with heart failure (37). However, few studies, to our knowledge, described the effects of ECR programs on exercise tolerance of end-stage heart failure patients awaiting heart transplantation (11, 16). The goal of our study was to evaluate the ECR program’s effects on the preoperative exercise capacity of a heart transplant candidate. Our main result showed that the ECR program increased the exercise tolerance of our end-stage heart failure patient. We also found a disassociation in the training responses of $\dot{\text{V}}$O_2_ peak and $\dot{\text{V}}$_E_/$\dot{\text{V}}$CO_2_ slope. First, we are going to discuss the effects of the ECR program on resting heart responses and NT-proBNP values.

Plasma NT-proBNP values are widely used in clinical practice to evaluate the severity of heart failure. While NT-proBNP may serve as an indicator of exercise tolerance in chronic heart failure (38), a previous systematic review reported beneficial effects of aerobic exercise training on NT-proBNP in patients with heart failure who performed a similar training to our subject (39). A tight relationship was found between NT-proBNP and resting HR in the elderly (40). In total, 60 sessions of our ECR program based on French national recommendations induced an improvement in NT-proBNP associated with a decrease in resting HR in our patient. It was established that the decrease in resting HR was inversely associated with cardiovascular morbidity and mortality (41). However, our finding should be considered with caution. It was recommended to measure resting HR in the supine position (42), but in our study, the resting HR measurement was carried out in a sitting position just before the CPET. Two important cardiac predictors of exercise capacity, i.e., the TAPSE and the cardiac index, were improved with the ECR program and approached normal values. A significant correlation was previously found between NT-proBNP and TAPSE in 60 patients with chronic heart failure (43). Furthermore, Nakano et al. observed a significant positive correlation between TAPSE and cardiac index in patients (44).

It was therefore not surprising to observe the $\dot{\text{V}}$O_2_ peak improvement (+ 71.3%) and the increased $\dot{\text{V}}$O_2_ at the anaerobic threshold in our patient. Gimeno-Santos et al. recently showed the beneficial effects of 16 exercise sessions (i.e., two times a week throughout an 8-week training program) on $\dot{\text{V}}$O_2_ peak (+ 27.0 ± 45.0%) in 11 patients placed on a heart transplantation list (16). Troisi et al. (43) observed that chronic heart failure patients with TAPSE greater than 16.0 mm had higher $\dot{\text{V}}$O_2_peak and $\dot{\text{V}}$O_2_ at AnTh. TAPSE was independently and significantly associated with $\dot{\text{V}}$O_2_peak, whereas resting LVEF was not (45). Together, these results suggested that the increase in exercise capacity with the ECR program, supported by the increased $\dot{\text{V}}$O_2_peak and submaximal $\dot{\text{V}}$O_2_ at AnTh, was partly due to structural and functional cardiac modifications without obvious improvements in resting LVEF. This could also explain the disassociation between $\dot{\text{V}}$O_2_peak and ventilatory efficiency responses to the exercise-based cardiac rehabilitation in our patient. The cardiac index and TAPSE increase observed from T_0_ to T_2_ provide further evidence for the positive exercise training structural and functional effects on the cardiovascular system in our end-stage heart failure patient awaiting transplantation as previously observed (46, 47). Giallauria et al. previously showed a significant correlation between the changes in NT-proBNP at rest and LV volumes after 6 months of exercise training in cardiac patients with moderate LV dysfunction (48). Then, the improvement in cardiac function associated with a slight decrease in plasma NT-proBNP concentration would have later resulted in an increase in $\dot{\text{V}}$O_2_peak associated with a delayed $\dot{\text{V}}$_E_/$\dot{\text{V}}$CO_2_ slope decrease (48, 49).

Finally, our results showed that the ECR program induced an increase in peak HR, which was associated with an HR decrease at rest. The difference between peak exercise and resting HR, called HR reserve (HRR), was classically used to evaluate chronotropic responses (50). It was recommended to measure resting HR in a supine position (42). The measurement of HR recovery could be an alternative solution. Defined as the difference between peak HR and HR obtained 1 min after exercise cessation (51), the HR recovery is however, dependent on post-exercise recovery modalities (52, 53). In the present study, HR recovery was probably affected by the 3-min unloaded pedaling of post-exercise recovery. Thus, it would be speculative in the current study to estimate HRR and HR recovery and conclude that the ECR program resulted in favorable changes in chronotropic function for our heart transplant candidate based simply on our HR measurements.

## Conclusion

Despite some methodological limitations, our results, taken together, may explain the improvement in the Seattle Heart Failure Model score. Therefore, we can conclude that the improvement in exercise capacity and cardiorespiratory function following ECR helped delay the heart transplant surgery in our patient on the transplant waiting list.

### Patient Outcome

Our patient continued to perform regular physical exercise until the first COVID19 lockdown when he completely stopped any form of physical activity. In January 2022, he was hospitalized for septic and cardiogenic shocks and died several days later.

## Data Availability Statement

The raw data supporting the conclusions of this article will be made available by the authors, without undue reservation.

## Ethics Statement

Written informed consent was obtained from the relevant individual for the publication of any potentially identifiable images or data included in this article.

## Author Contributions

P-ML, AP, YG, and FK contributed to conception and design of the study. FK and YG organized the database. AP and P-ML wrote the first draft of the manuscript. PL reviewed and edited all sections of the manuscript. All authors contributed to manuscript revision, read and approved the submitted version.

## Conflict of Interest

The authors declare that the research was conducted in the absence of any commercial or financial relationships that could be construed as a potential conflict of interest.

## Publisher’s Note

All claims expressed in this article are solely those of the authors and do not necessarily represent those of their affiliated organizations, or those of the publisher, the editors and the reviewers. Any product that may be evaluated in this article, or claim that may be made by its manufacturer, is not guaranteed or endorsed by the publisher.
